# Upregulation of COPB2 Promotes Prostate Cancer Proliferation and Invasion Through the MAPK/TGF-β Signaling Pathway

**DOI:** 10.3389/fonc.2022.865317

**Published:** 2022-05-06

**Authors:** Yanyan Feng, Chuanyu Sun, Lifeng Zhang, Hongyuan Wan, Hangsheng Zhou, Yongquan Chen, Lijie Zhu, Guowei Xia, Yuanyuan Mi

**Affiliations:** ^1^ Wuxi Medical College, Jiangnan University, Wuxi, China; ^2^ Department of Urology, Affiliated Hospital of Jiangnan University, Wuxi, China; ^3^ Department of Urology, Huashan Hospital, Fudan University, Shanghai, China; ^4^ Department of Urology, Affiliated Changzhou No. 2 People’s Hospital of Nanjing Medical University, Changzhou, China

**Keywords:** COPB2, MAPK/TGF-β signal pathway, NUPR1, proliferation, invasion, prostate cancer

## Abstract

There is increasing evidence that coatomer protein complex subunit beta 2 (COPB2) plays an important role in various cancer types. This study explored the role and the downstream mediators of *COPB2* in prostate cancer (PCa). The expression of COPB2 was determined by the Cancer Genome Atlas database and enzyme-linked immunosorbent assay. COPB2 expression was upregulated in PCa tissues and correlated with Gleason score, biochemical recurrence, and poor prognosis. The functional roles of *COPB2* in PCa were verified through a series of experiments. Knocking down COPB2 expression inhibited the growth and clonogenesis of PCa cells, promoted cell apoptosis, and inhibited the ability of scratch repair, invasion of PCa cells, and tumor growth in Nude mice. To analyze downstream signaling pathways, ingenuity pathway analysis, GSEA, and whole-genome expression spectrum GeneChip analysis were used. Western blot revealed that COPB2 expression promoted the proliferation and invasion of PCa cells by regulating the MAPK/TGF-β signaling pathway. The interacting protein (nuclear protein 1, NUPR1) was identified *via* Co-IP, real-time PCR, Western blot, and TCGA database in sampled tissues. The expressions of the interaction proteins NUPR1 and COPB2 were negatively regulated by each other. *COPB2* could be a new biomarker for PCa diagnosis and monitoring and to provide a theoretical basis for identifying effective drug intervention targets through in-depth mechanistic studies.

## Introduction

Approximately 248,530 new cases of prostate cancer (PCa) were diagnosed in the United States in 2021, causing 34,130 deaths in the same year. (1) With the economical and sociocultural development, the expended life expectancy and westernized lifestyle led to the increasing incidence and mortality rates of PCa in China (2, 3). At present, PCa is ranked sixth among the most frequent cancers and tenth among the most common cause of cancer-related deaths in China (2, 4–6).

Androgens are necessary for the occurrence and development of PCa; therefore, androgen deprivation therapy (ADT) is regarded as a first-line therapy for local/locally advanced/local metastatic PCa (7). Nevertheless, most patients develop castration-resistant PCa (CRPC) after 2 to 3 years of ADT, leading to bone metastasis in over 90% of patients (8, 9). Novel targeted drugs have been developed. These include abiraterone, which blocks the cytochrome p450 17A1 enzyme; cabazitaxel, which targets tubulin; sipuleucel-T, which targets the immune system; and enzalutamide, which inhibits the nuclear translocation, DNA binding, and coactivator recruitment of androgen receptors (10, 11). However, metastatic CRPC is still considered an incurable disease (12). Hence, there is an urgent need to identify unreported biomarkers and related molecular mechanisms underlying the development of CRPC.

To identify potential biomarkers or drug targets for PCa/CRPC, the differential expression of COPB2 was detected by label-free quantitative proteomics (13). *COPB2* is situated on chromosome 3q23 and encodes a protein containing 906 amino acids (102.5 kDa) (14, 15). Furthermore, COPB2 is not only involved in vesicle transport, but also participates in the development of tumor cells as an oncogene (15). *COPB2* was also proved to be involved in PCa cell proliferation, cycle, and apoptosis *in vitro* (13, 16). To further explore whether *COPB2* also participates in cell invasion and to assess its underlying role and mechanism of action, clinical analysis using cell culture models and Nude-mice models and GeneChip analysis were applied. In summary, investigating the role of *COPB2* in PCa/CRPC development is critical for determining whether it may serve as a potential biomarker in future medical research.

## Materials and Methods

### Ethical Clearance

Paraffin-embedded tissue blocks were obtained from the department of pathology, the Affiliated Hospital of Jiangnan University, Affiliated Changzhou No. 2 Hospital of Nanjing Medical University and Huashan Hospital of Fudan University between January 2012 and December 2016, which included samples from 118 PCa patients. None of the patients had received ADT or radiotherapy before surgery. Clinicopathological and prognostic parameters are shown in **Table 1**. The clinical samples and patients (LS202128) were approved by the Research Ethics Committee of Affiliated Hospital of Jiangnan University, Affiliated Changzhou No. 2 Hospital of Nanjing Medical University and Huashan Hospital of Fudan University.

**Table 1:** Correlation between COPB2 expression and clinicopathological factors.

Covariates	Total (%)	COPB2 expression		P-value	Statistic method
		Low (%)	High (%)		
**Age (years)**					
** <70**	63 (53.39)	46 (63.89)	17 (36.96)		chisq
** >=70**	55 (46.61)	26 (36.11)	29 (63.04)	**0.007**	chisq
**BMI**					
** <25**	74 (62.71)	44 (61.11)	30 (65.22)		chisq
** >=25**	44 (37.29)	28 (38.89)	16 (34.78)	0.798	chisq
**PSA value (initial)**					
** <10**	41 (34.75)	29 (40.28)	12 (26.09)		chisq
** >=10**	77 (65.25)	43 (59.72)	34 (73.91)	0.167	chisq
**Gleason score**					
** <=7**	72 (61.02)	56 (77.78)	16 (34.78)		chisq
** >7**	46 (38.98)	16 (22.22)	30 (65.22)	**<0.001**	chisq
**TNM stage**					
** T1-T2**	70 (59.32)	42 (58.33)	28 (60.87)		chisq
** T3-T4**	48 (40.68)	30 (41.67)	18 (39.13)	0.935	chisq
**Biochemical recurrence**					
** Negative**	98 (83.05)	68 (94.44)	30 (65.22)		fisher
** Positive**	20 (16.95)	4 (5.56)	16 (34.78)	**<0.001**	fisher

Bold values mean P < 0.05.

### Immunohistochemistry Staining

Paraformaldehyde (4%) was used to fix collected tissue samples. Paraffin-embedded tissues were cut into 4-μm-thick sections. Tissue sections were dewaxed, hydrated, then permeabilized with 0.5% Triton X-100 at room temperature (25°C) for 10 min, blocked with 3% H_2_O_2_ for 30 min and 10% goat serum for 30 min. Next, the samples were incubated with antibodies against COPB2 (1:100 dilution, Bioworld, Minnesota, USA) at 4°C overnight. Thereafter, images of the sections were taken. The subsequent steps were performed using the GTVision III Detection System/Mo&Rb (Gene Tech, Shanghai, China).

### Cell Lines and Cultures

Two PCa cell lines, PC-3 and DU-145, were purchased from the Cell Bank of the Chinese Academy of Sciences (Shanghai, China) and cultured in RPMI-1640 medium (Gibco, USA) containing 10% fetal calf serum (Gemini, USA), 100 μg/ml streptomycin (Sangon Co. Ltd., Shanghai, China), and 100 U/ml penicillin (Sangon Co. Ltd., Shanghai, China) at 37°C in a 5% CO_2_ incubator.

### Bioinformatics Analysis

*COPB2* expression was collected and downloaded from The Cancer Genome Atlas (TCGA) online database (http://tcga-data.nci.nih.gov/tcga/). First, the expression of *COPB2* in normal and cancerous prostate tissues was analyzed. Second, the biochemical recurrence based on prostate-specific antigens was evaluated using the Kaplan–Meier survival curve. In addition, to assess the association between *COPB2* expression and disease progression (such as Gleason score and metastasis), *COPB2* expression was investigated according to the Gleason score (≤ 7)/(> 7) in primary/metastatic PCa; for that, publicly available datasets from the Gene Expression Omnibus (GEO, http://www.ncbi.nlm.nih.gov/geo/) were used. All TCGA and GEO data were calculated and processed using R software (http://www.r-project.org) and SPSS 17.0.

### Serum Preparation and Monitoring of Secretory COPB2 Expression Using Enzymelinked Immunosorbent Assay (ELISA)

Blood samples were obtained from 200 pairs of PCa patients and controls. Whole-blood samples were promptly centrifuged at 1000 × *g* for 5 min, and the supernatant was collected and stored at −20°C. Serum *COPB2* levels were determined by a commercially available ELISA kit (Human COPB2 ELISA kit, Biomart, Shanghai, China), according to the manufacturer’s instructions. Briefly, serum specimens, standards, and biotin conjugates were added to the wells and incubated for 1 h at 37°C. The unbonded material was then washed away. After adding chromogen solution, the samples were incubated at 37°C for 15 min in the dark. The colorless solution was transformed into a blue solution, and the color intensity was proportional to the COPB2 content in the sample. Owing to the action of the acidic stop, the color turned yellow. Staining reaction products were measured at a wavelength of 450 nm using an automated ELISA analyzer (Rayto, RT-1904C Chemistry Analyzer, Atlanta, GA, USA).

### Quantitative Real-Time PCR

RNA extraction from PCa cell lines was performed using the RNAiso Plus kit (Takara, Japan). RNA was then reverse-transcribed into cDNA using the PrimeScript™ RT reagent kit (Takara) according to the manufacturer’s instructions. The primer sequences used for *COPB2*, *NUPR1* and *GAPDH* genes were shown in **Table 2**. SYBR Green Real-Time PCR assay kit (Takara) was used for PCR with an ABI 7500 PCR System (ABI, Co. Ltd., USA). The qRT-PCR protocol consisted of: initiation at 95°C for 30 s, followed by 40 cycles at 95°C for 5 s and 59°C for 30 s. The relative mRNA expression (*COPB2*/*GAPDH* or *NUPR1*/*GAPDH*) was determined using the 2^-ΔΔCt^ method. All analyses were conducted in triplicate.

**Table 2:** Sequences of primers used for qPCR.

mRNA	Primer	
	FORWARD	REVERSE
COPB2	5′-GTGGGGACAAGCCATACCTC-3′	5′-GTGCTCTCAAGCCGGTAGG-3′
NUPR1	5′-ATAGCCTGGCCCATTCCTAC-3′	5′-GCAGCAGCTTCTCTCTTGGT-3′
GAPDH	5′-TGACTTCAACAGCGACACCCA-3′	5′-CACCCTGTTGCTGTAGCCAAA-3′

### Lentivirus-Mediated Small Hairpin RNA Construction and Determination of Infection Efficiency

shRNA target sequence (AGATTAGAGTGTTCAATTA) for COPB2 gene (NM_004766.2, http://www.ncbi.nlm.nih.gov/nuccore/NM_004766.2) was designed, and a non-silencing shRNA sequence (TTCTCCGAACGTGTCACGT) was adopted as a negative control (13, 16). Additionally, the shRNA target sequence (CCAAGCTGCAGAATTCAGA) for *NUPR1* was obtained from Gene Chem Company (Shanghai, China). The process of detecting the infection efficiency was the same as that in previous studies (13, 16). The efficiency of transfection was > 80% *via* green fluorescent protein (GFP) fluorescence.

### Cell Proliferation and Colony-Formation Assays

The proliferation of PC3 and DU145 cells was detected by MTT assay. After achieving the logarithmic growth phase, both of shCOPB2 and shCtrl cell cultures were trypsinized and resuspended in RPMI-1640 medium. The cells were plated in five 96-well plates followed by incubation at 37°C and 5% CO_2_. After incubation for 1, 2, 3, 4, and 5 days, the cells were incubated with 10 μl MTT for 4 h. Finally, the optical density at a wavelength of 570 nm was measured. According to the data, statistical data mapping and construction of cell proliferation curves were further analyzed. This assay was repeated in triplicate.

Cell collection and plate laying were the same as that in the MTT assay. Cellomics was performed 1, 2, 3, 4 and 5 days after plate laying. By adjusting the input parameters, cells transfected with lentivirus (the lentivirus included a plasmid of GFP) in each well were counted, and the data were statistically plotted.

For the colony-formation assays, cells were seeded in six-well plates; each experimental group was plated in three wells. The cells were cultured and observed for 10 days, and the medium was changed every 3 days. The colonies were fixed with paraformaldehyde (1 ml/well; Shanghai Pharmaceutical Group, Shanghai, China) for 30–60 min followed by Giemsa staining (500 ml/well; Shanghai Sangong, China) for 20 min. The colonies were visualized and counted using a fluorescence microscope (Olympus, Japan). This assay was performed in triplicate.

### Apoptosis Analysis

Flow cytometry (FCM) was used to determine the apoptosis rate. shCOPB2 and shCtrl cell cultures were trypsinized and resuspended in RPMI-1640 medium after achieving the logarithmic growth phase. The cells were plated in 6-well plates, harvested, washed with phosphate-buffered saline, and centrifuged for 5 min. The cells were then washed with 1× binding buffer and centrifuged for 3 min. Cell pellets were resuspended in 200 μl 1× binding buffer. The suspension was stained with 5-ul-Annexin V-PE and 5-ul-7-AAD in a binding buffer in the dark at room temperature (25°C) for 15 min, then the cells were incubated for 1 h. The cells were analyzed using a flow cytometer (Thermo, USA) within 1 h to determine the proportion of cells in each phase of the cell cycle in each group and the apoptosis rate. This assay was performed in triplicate.

### Western Blot Analysis

The expression of proteins was analyzed by western blot. Briefly, total protein was extracted using an immunoprecipitation protein lysis buffer (Beyotime Biotechnology, Shanghai, China). The protein concentration was determined using a BCA protein determination kit (Vazyme, Nanjing, China). Protein was separated by SDS-polyacrylamide gel electrophoresis. Each 100 μg protein sample was loaded onto 10% SDS-Page gel and transferred to a PVDF membrane (Amersham Biosciences, Buckinghamshire, UK) for 100 min. The primary antibodies were incubated in blocking buffer overnight at 4°C. The primary antibodies used were as follows: anti-GAPDH (1:3000 dilution; Bioworld, USA), anti-cyclin-dependent kinase 2 (CDK2; 1:1000 dilution; Cell Signaling Technology, USA), anti-cyclin D1 (1:1000 dilution; Cell Signaling Technology, USA), anti-CDK4 (1:1000 dilution; Cell Signaling Technology, USA), anti-p21 Waf1/Cip1 (1:1000 dilution; Cell Signaling Technology, USA), and anti-p27 Kip1 (1:1000 dilution; Cell Signaling Technology, USA). anti-MEK (1:1000 dilution; Cell Signaling Technology, USA), anti-p-MEK (1:1000 dilution; Cell Signaling Technology, USA), anti-ERK (1:1000 dilution; Cell Signaling Technology, USA), anti-p-ERK (1:1000 dilution; Cell Signaling Technology, USA), anti-P38 (1:1000 dilution; Cell Signaling Technology, USA), anti-p-P38 (1:1000 dilution; Cell Signaling Technology, USA), anti-MMP2 (1:1000 dilution; Cell Signaling Technology, USA), anti-MMP9 (1:1000 dilution; Cell Signaling Technology, USA), anti-JUK (1:1000 dilution; Cell Signaling Technology, USA), anti-p-JUK (1:1000 dilution; Cell Signaling Technology, USA), anti-E2F1 (1:1000 dilution; Cell Signaling Technology, USA), anti-PCNA (1:1000 dilution; Cell Signaling Technology,USA), anti- ZEB1 (1:1000 dilution; Cell Signaling Technology, USA), anti-transforming growth factor (TGF)-β (1:1000 dilution; Cell Signaling Technology, USA). Then secondary antibodies were incubated with fluorescently labeled for 2 h at room temperature (25°C). The intensity of the bands was quantified using the Image Lab™ Software (Bio-Rad). Bands were visualized using an Odyssey detection system (Licor Biosciences, Nebraska, USA). Image J was used for densitometry analysis. This assay was performed in triplicate.

### Wound Healing Assay

shCOPB2 and shCtrl cells were cultured in RPMI-1640 medium with 10% fetal bovine serum. Wounds were made in the cell monolayer using a 10 μl plastic pipette tip. The size of the wound was imaged and measured after 12 h of wound formation. The cell migration area was measured with dashed areas and normalized to control cells. This assay was performed in triplicate.

### Cell Invasion Assay

Cell invasion was evaluated using Transwell assays; the Transwell chambers (pore size: 8 μm, Corning) were matrigel-coated. The matrix glue was diluted with serum-free medium at 1:5. The lower chamber was filled with 500 μL of 20% FBS medium. Transfected PC3 and DU145 cells (6×10^4^) in 200 μL of serum-free medium were gently loaded onto each filter insert (upper chamber) and then incubated at 37°C for 48 h. The filter inserts were removed from the chambers, fixed with 500 μL 4% paraformaldehyde for 40 min and stained with hematoxylin for 20 min. The samples were subsequently washed, dried, and mounted onto slides. The migratory cells were stained blue, visualized under an inverted microscope, and then counted in five random fields for statistical analysis.

### Tumor Formation Assay in a Nude-Mouse Model

Virus-infected cells in the logarithmic phase at a density of 5×10^6^/ml were mixed with 5 mg/ml basement membrane matrix to prepare a 1 ml cell suspension. A volume of 0.8 ml cell suspension containing 4×10^6^ cells was injected into the right cutaneous axilla of Nude mice. Tumor size was observed and recorded every 4 days, when tumor length and diameter were measured. After 24 days, the Nude mice were euthanized by cervical pulp dissection, and the tumor was completely removed. Finally, the tumor growth curve was plotted. The animal study (JN. No20201030c0561225[274]) was reviewed and approved by the Ethics Review Committees of the Jiangnan University.

### GeneChip Analysis

Total RNA was extracted using the TRIzol method. RNA concentrations were determined with a NanoDrop 2000 spectrophotometer (Thermo Scientific, Pittsburgh, PA, USA). Total RNA from were screened for differentially expressed genes using an Agilent RNA 6000 Nano Kit (Agilent, Santa Clara, CA, USA), and a PrimeView Human GeneChip (Agilent, Santa Clara, CA, USA) was used for microarray analysis. RNA labelling and hybridisation to Agilent miRNA microarray chips were performed with a GeneChip Hybridization Wash and Stain Kit (Agilent, Santa Clara, CA, USA) (17, 18). The GeneChip data was used to analyze by gene set enrichment analysis (GSEA), and differentially expressed genes were annotated using ingenuity pathway analysis (IPA) to predict path changes.

### Co-Immunoprecipitation (Co-IP) Assay

This experiment was performed using 293T cells. An appropriate amount of COPB2 primary antibody was added to the cell lysate to couple the antibody to Protein A sepharose. According to the amount of expressed proteins and cell lysate, 1–5 μg antibody and 10 μl Protein A (50% suspension) were mixed to obtain a total volume of 1 ml, and the mixture was incubated at 4°C for 4 h. Beads were deposited by centrifugation (centrifugation at 10,000 rpm for 30 s) and washed three times with 1×HNTG buffer. The precipitate was eluted with 30 μl 1× Laemmli loading buffer. The binding of *COPB2* and *NUPR1* was detected *via* sodium dodecyl sulfate–polyacrylamide gel electrophoresis and western blot after heating at 100°C for 10 min. In turn, *NUPR1* was first added, and COPB2-bound *NUPR1* was detected.

### Statistical Analysis

Before performing statistical analyses, the distribution of all data was normalized. The Wilcoxon nonparametric test was used to compare the expression of COPB2 in the serum. Fisher’s exact test or chi-square test was used to analyze the correlation between *COPB2* and clinical features and prognosis of PCa. Univariate and multivariate analyses of COPB2 expression and clinical features and prognosis of PCa were performed using binary logistic regression. The *t-*test was used for comparison between groups. Statistical analysis was performed using SPSS17.0 software, and *P*-values < 0.05 were considered statistically significant. Data are expressed as the mean ± standard deviation. Images were produced using GraphPad Prism 8 and Illustrator CC 2018.

## Result

### COPB2 Expression is Upregulated in PCa Tissues and Correlated With Poor Prognosis

**Figure 1:**
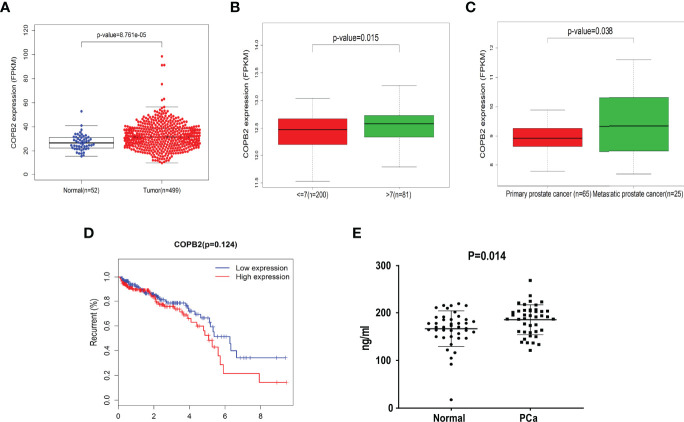
Difference expression of *COPB2* in clinical characteristics of PCa. **(A)** Expression of COPB2 between PCa tissue (499) and normal tissue (52) from TCGA cohort. **(B)** Level of COPB2 expression was analyzed in Gleason score ≤ 7 (200) and Gleason score > 7 (81) from GEO dataset (GSE16560). **(C)** Level of COPB2 expression was analyzed in primary PCa samples (65) and metastatic PCa samples (25) from GEO dataset (GSE6919). **(D)** An analysis of recurrence-free survival in patients with PCa by the Kaplan-Meier method. FPKM: Fragments Per Kilobase of exon model per Million mapped fragments. **(E)** The secretory of *COPB2* in serum from peripheral blood between normal samples and PCa patients.

The data (**Figures 1A-D**) were derived from TCGA. First, the expression of COPB2 in PCa tissues (n = 499) and normal tissues (n = 52) from TCGA cohorts were analyzed. According to **Figure 1A**, COPB2 was overexpressed in PCa tissues (*P* < 0.01). Second, the level of COPB2 expression was higher in patients with Gleason score > 7 than in patients with Gleason score ≤ 7 (200) from the GEO dataset (GSE16560*, P* = 0.015) (**Figure 1B**). Next, COPB2 expression was higher in primary PCa samples (65) than in metastatic PCa samples (25) from the GEO dataset (GSE6919) (**Figure 1C**). The curve and correlation analyses showed a tendency for patients with high COPB2 expression to have a higher percentage of biochemical recurrence (**Figure 1D**). These databases indicated that *COPB2* was closely associated with PCa, and further clinical data were needed to verify the clinical relevance of *COPB2*. According to the samples and information collected from 118 patients with PCa who underwent radical prostatectomy (RP), the correlation between COPB2 expression level, clinical characteristics, and biochemical recurrence of PCa were analyzed. The results showed that COPB2 expression level was positively correlated with Gleason score and postoperative biochemical recurrence (*P* = 0.007, *P* < 0.001, and *P* < 0.001, respectively) (**Table 1**). Univariate and multivariate analyses suggested that patients with high COPB2 expression had a higher postoperative biochemical recurrence rate (*P* = 0.007 and 0.012, respectively) (**Table 3**). Peripheral blood samples were collected from 42 PCa patients and 42 benign prostate hyperplasia (BPH) patients. Serum was collected by centrifugation, and an ELISA kit was used to detect secreted *COPB2*. The results showed that the content of secreted *COPB2* in the peripheral blood and serum of PCa patients was significantly higher than in BPH patients. (*P* = 0.014, **Figure 1E**).

**Table 3:** Prognostic factors for recurrence-free survival in univariate and multivariate analyses.

Covariates	Univariate analysis		Multivariate analysis	
	HR (95%CI)	P	HR (95%CI)	P
**Age**	1.218 (0.481-3.087)	0.677	0.895 (0.334-2.400)	0.825
**BMI**	1.510 (0.609-3.747)	0.374	1.408 (0.518-3.831)	0.503
**PSA**	1.415 (0.508-3.943)	0.507	0.954 (0.331-2.836)	0.933
**Gleason score**	2.073 (0.817-5.256)	0.125	0.859 (0.274-2.692)	0.794
**Tumor stage**	1.778 (0.701-4.509)	0.226	1.990 (0.721-5.487)	0.184
**COPB2 expression**	4.697 (1.535-14.370)	**0.007**	5.443 (1.443-20.530)	**0.012**

Bold values mean P < 0.05.

### Function of *COPB2* in the DU-145 Cell Line

**Figure 2:**
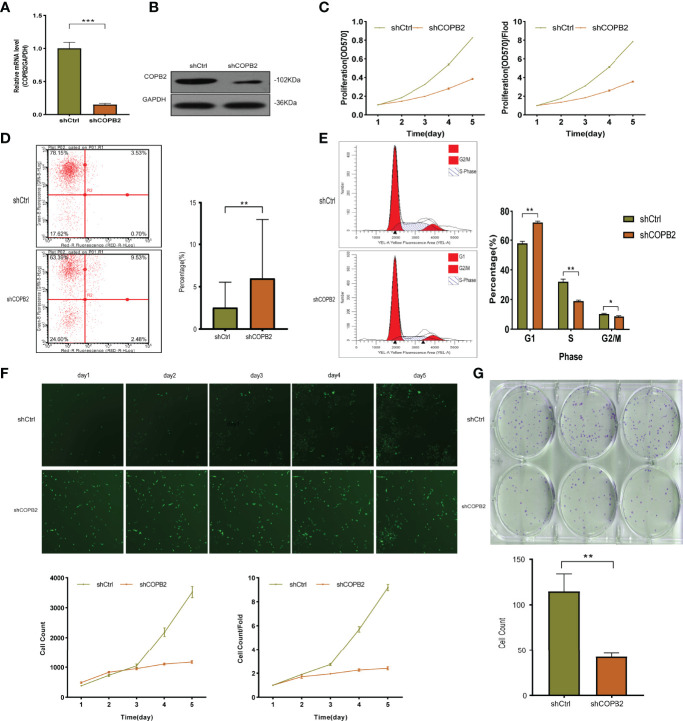
The function of COPB2 protein in DU-145 cell line. **(A)** Identification of *COPB2* knockdown efficiency using shCOPB2 by qRT-PCR. **(B)** Western blot analysis of COPB2 protein expression in shCtrl and shCOPB2 groups. **(C)** Cell proliferation was assessed in shCtrl and shCOPB2 groups using MTT assays. **(D)** Cell apoptosis: FCM showed that the shCOPB2 increased cell apoptosis compared with the shCtrl group. **(E)** Cell cycle: FCM analysis shCOPB2 induced a G1-phase arrest. An obvious increase in the G1-phase cell population and a significant decrease in the S-phase and G2-phase cell population were observed compared with the shCtrl group. **(F)** Cell proliferation was significantly inhibited in shCOPB2 group compared with shCtrl group according to GFP-based Cellomics Array Scan VTI imaging. **(G)** Colony-formation analysis by light microscopy. After lentiviral transfection of DU-145 cells, the shCOPB2 group displayed a significantly reduced number of cell colonies compared with the shCtrl group. *P < 0.05, **P < 0.01, ***P < 0.001.

The expression of COPB2 knockdown in DU145 cells was significantly inhibited, as indicated by qRT-PCR and western blot, compared to COPB2 expression in the shCtrl group (**Figures 2A, B**). MTT assay illustrated that the proliferation abilities of PCa cells were reduced notably after *COPB2* knockdown (**Figure 2C**). FCM showed that knocking down *COPB2* increased cell apoptosis compared to that in the shCtrl group (**Figure 2D**). Moreover, the effects of *COPB2* knockdown on the cell cycle of PCa cells were confirmed. The results showed that *COPB2* knockdown resulted in significantly decreased ratios of S phase and G2/M phase arrest (**Figure 2E**). According to GFP-based Cellomics Array Scan VTI imaging, cell proliferation was significantly inhibited in the shCOPB2 group compared with shCtrl group (**Figure 2F**). Colony-formation assay testified the colony-formation abilities of PCa cells were reduced clearly after *COPB2* knockdown (**Figure 2G**). Overall, these results demonstrated that silencing *COPB2* inhibited PCa cell proliferation and promoted apoptosis.

### *COPB2* Knockdown Inhibits the Ability of Wound Healing and Invasion in PC-3 and DU-145 Cell Lines

**Figure 3:**
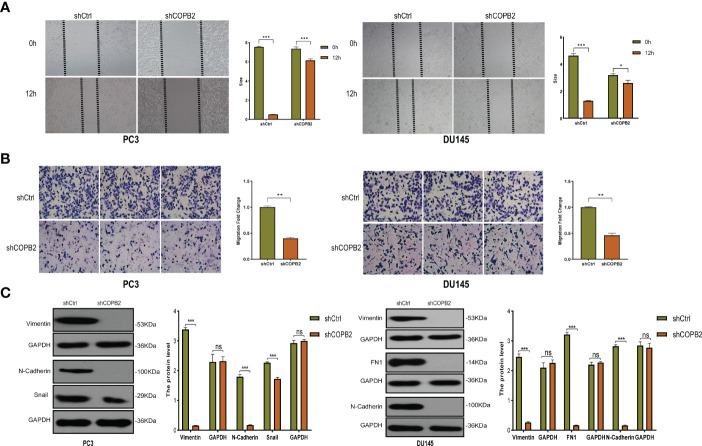
*COPB2* knockdown inhibited migration and invasion in PC-3 and DU-145 cell lines. **(A)** A wound healing assay was performed to study the migration of COPB2 knockdown on PC-3 and DU-145 cell. **(B)** A Transwell assay was performed to study the invasion of *COPB2* knockdown on PC-3 and DU-145 cell. **(C)** Motility inhibition by *COPB2* knockdown was associated with EMT suppression in PC-3 and DU-145 cells. EMT markers (Vimentin, N-Cadherin, Snail, and FN1) were assessed by western blot. *P < 0.05, **P < 0.01, ***P < 0.001, ns, not significant.

The effect of *COPB2* on PCa metastasis was explored. The shCOPB2 group had decreased wound size compared to the shCtrl group in wound healing assays in PC3 and DU145 cells lines (**Figure 3A**). Transwell assays showed that *COPB2* knockdown inhibited the invasion of PC3 and DU145 cells (**Figure 3B**). Western blot was used to detect the epithelial-mesenchymal transformation (EMT) proteins associated with cell invasion. The results showed that the expression of EMT-related interstitial proteins (Vimentin, N-cadherin, Snail, and FN1) was significantly decreased in the shCOPB2 group (**Figure 3C**), suggesting that *COPB2* knockdown significantly inhibited EMT transformation and tumor invasion.

### *COPB2* Knockdown Inhibits Tumor Growth *in Vivo*

**Figure 4:**
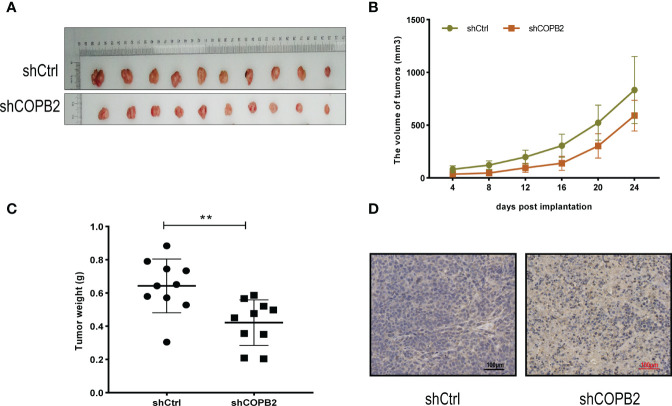
*COPB2* knockdown inhibited tumor growth in Nude mice. **(A)** Tumors grew in Nude mice were removed at day 24 after xenograft. **(B, C)** Knockdown of *COPB2* inhibited tumor growth of xenograpted PC-3 cells based on two-dimensional caliper measurements of volume and weight of tumors, respectively. **(D)** Representative immunostaining images (original magnification, ×100). **P < 0.01.

For investigating the function of *COPB2 in vivo*, a tumor xenograft mouse model was constructed using PC3 cells, and the tumor size was measured every 4 days. After 24 days, the tumor was dissected (**Figure 4A**). The results showed that the growth rate of shCOPB2 group was significantly slower (*P* < 0.05, **Figure 4B**), and the corresponding tumor weight was significantly lower than shCtrl group (*P* < 0.05, **Figure 4C**). In addition, IHC staining indicated that COPB2 expression in the shCOPB2 group was significantly lower than shCtrl group in tumor tissues (**Figure 4D**). Together, these data confirmed that *COPB2* knockdown inhibited PCa tumor growth *in vivo*.

### Cell Cycle and Apoptosis-Related Proteins Were Detected *via* Western Blot

**Figure 5:**
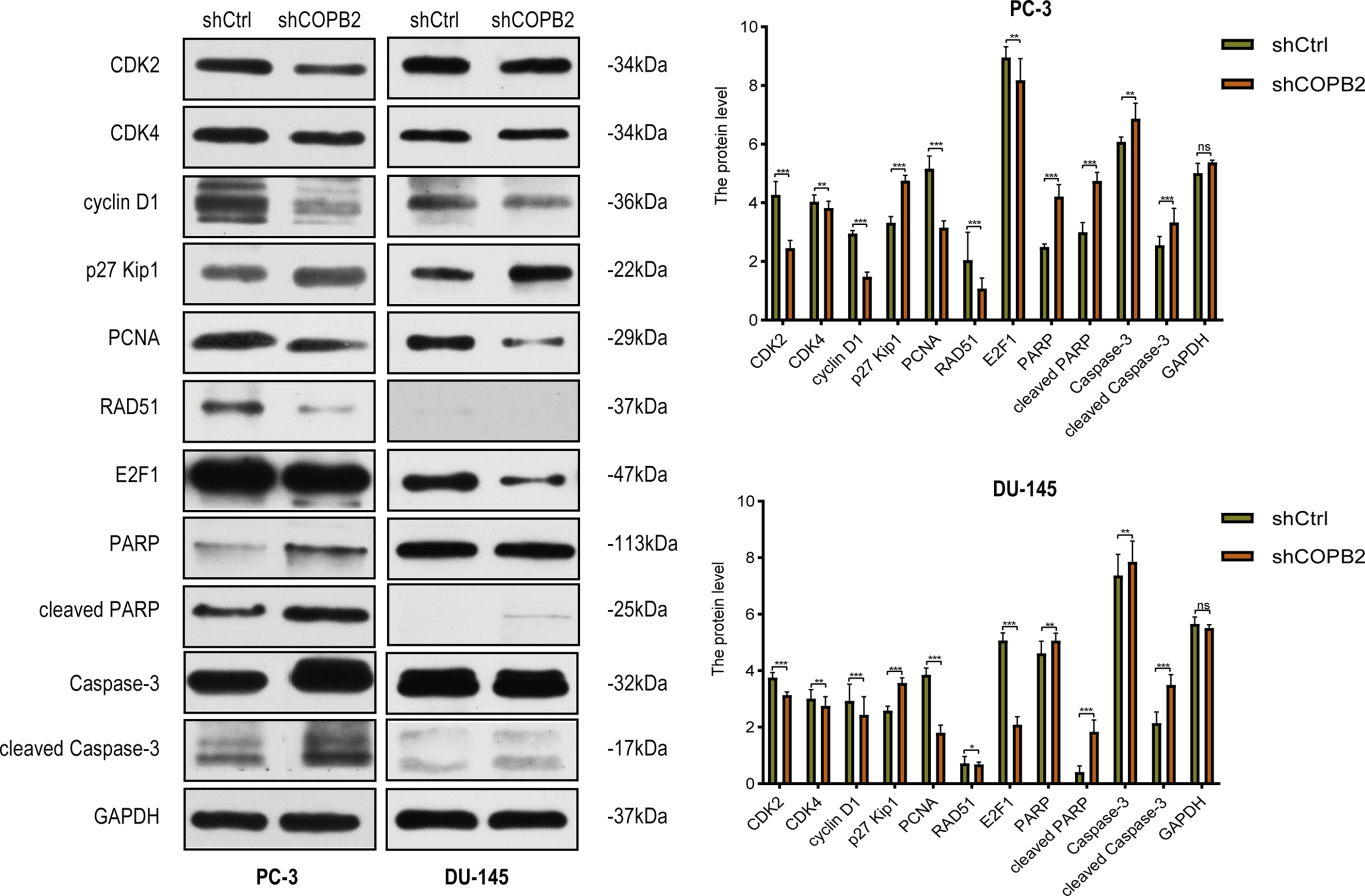
Cell cycle and apoptosis signaling pathways mediated COPB2-induced proliferation of PC-3 and DU-145 cells. Western blot analysis of CDK2, CDK4, cyclin D1, p27 Kip1, PCNA, RAD51, E2F1, PARP, cleaved PARP, Caspase-3 and cleaved Caspase-3 in shCOPB2 and shCtrl groups in two PCa cell lines. *P < 0.05, **P < 0.01, ***P < 0.001, ns, not significant.

Cyclin-dependent proteins cyclin-dependent kinase (CDK2 and CDK4), cyclin D1, CDK inhibitor proteins (P27 kip1 and P21 WAF1/CIP1), PCNA, RAD51, and E2F1; apoptosis markers (PARP, cleaved PARP, caspase-3, and cleaved caspase-3) were detected *via* western blot. The results revealed that the expression of cyclin and cyclin-dependent kinase was reduced, and the expression of CDK inhibitor was increased in shCOPB2 group. Meanwhile, cleaved PARP and cleaved caspase-3 expression was significantly increased, suggesting that knocked-down *COPB2* blocked the cell cycle and promoted apoptosis, which was consistent with the results of functional experiments (**Figure 5**).

### Role of Increased COPB2 Expression in PCa and Downstream Mechanisms

**Figure 6:**
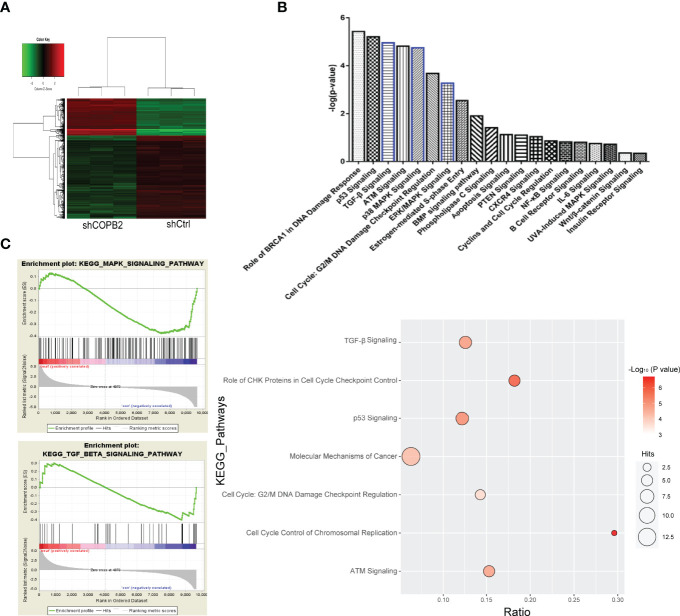
GeneChip analysis for *COPB2* knockdown and control groups, and IPA. **(A)** Hierarchical clustering graph of transcriptome expression of COPB2-knockdown samples and control samples in heatmap analysis. Pseudo-color was used to represent the intensity of gene expression from the array analysis. Green and red denote low and high expression, respectively. **(B)** IPA analyses the enrichment of downstream signaling pathways. **(C)** Correlation in enrichment plot, the horizontal axis represents genes, and the vertical line in the middle plot represents genes in this pathway. The ‘h’ (red) indicated that the pathway was enriched in the positively related pathway; the ‘l’ (blue) indicated that the pathway was enriched in the negatively related pathway.

COPB2 expression was interfered in PCa cell line PC-3, and the whole gene expression profile was detected by microarray. Genes with differential multiple selection (> 2 or < 0.5) were selected for IPA analysis combined with bioinformatics. Furthermore, heat map and hierarchal clustering of the transcriptome expression in COPB2-knockdown samples and control samples was analyzed. Pseudo-color was used to represent the strength of gene expression from the array analysis. Green denotes low expression, and red denotes high expression. According to the analysis of heat map, the gene-expression profile was different between shCOPB2 group and shCtrl group. (**Figure 6A**). In addition, IPA analysis of the enrichment of downstream signaling pathways indicated that mitogen-activated protein kinase (MAPK) and TGF-β signaling pathways, related to tumor proliferation and invasion, were ranked at the forefront of differential pathways (**Figure 6B**). To analyze microarray data by GSEA, and found that MAPK and TGF-β signaling pathways were enriched and positively correlated with COPB2 expression after *COPB2* knockdown. These results suggested that MAPK and TGF-β signaling pathways participated in the biological process of PCa proliferation and invasion as downstream regulatory signals of the COPB2. (**Figure 6C**). Differential genes were enriched by IPA, diseases and functions were classified. For instance, disease and function heat map showed the relationship between up/down regulated and differential gene expression and inhibition/activation in disease and function (**Supplementary Figure 1**); and disease and function histogram showed the enrichment of differentially expressed genes in disease and functional categories [-Log (*P*-value) Transformation] (**Supplementary Figure 2**). The upstream regulatory sub-network diagram showed the enrichment of differentially expressed genes in classical signaling pathways [-Log (P-value) Transformation] (**Supplementary Figure 3**). The regulatory effect network diagram showed the interaction between genes/regulators and corresponding functions in the dataset, and NUPR1 was the interaction protein downstream of COPB2 (**Supplementary Figure 4**).

### Confirmation of MAPK and TGF-β Signaling Pathways

**Figure 7:**
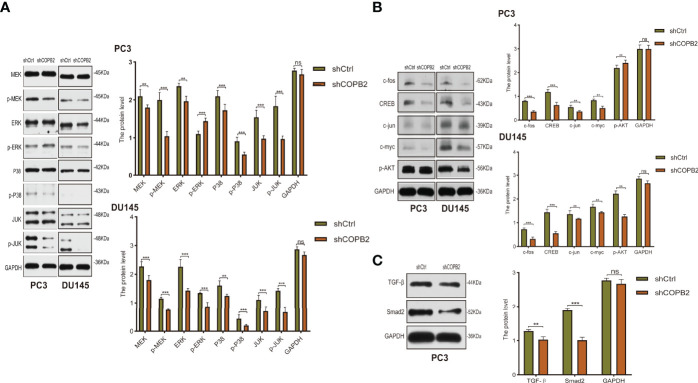
MAPK and TGF-β signaling pathways mediated COPB2-induced proliferation of PCa cells. **(A)** Western blot analysis of MEK. p-MEK, ERK, p-ERK, P38, p-P38, JUK, p-JUK in shCOPB2 and shCtrl groups in PC-3 and DU-145 cell lines. **(B)** Western blot analysis of c-fos, CREB, c-jun, and c-myc in shCOPB2 and shCtrl groups in PC-3 and DU-145 cell lines. **(C)** Western blot analysis of TGF-β and Smad2 in shCOPB2 and shCtrl groups in PC-3 cell lines. **P < 0.01, ***P < 0.001, ns, not significant.

Western blot analysis indicated that the expressions of the MAPK signaling pathway-related proteins p-MEK, p-P38, p-JUK, c-fos, CREB, c-jun, and c-myc were downregulated in the shCOPB2 group compared to shCtrl group (**Figures 7A, B**). The expression of p-ERK was inhibited in DU145 cells, nevertheless, activated in PC3 cells. It was suggested that *COPB2* knockdown led to changes in the MAPK signaling pathway. As MAPK signaling was involved in cell proliferation, *COPB2* might ultimately promote cell proliferation through downstream MAPK signaling. Second, we detected the expression of key proteins in the TGF-β signaling pathway (TGF-β and Smad2) in PC3 cells, and the results revealed that the expression of TGF-β and Smad2 was downregulated in the shCOPB2 group (**Figure 7C**), suggesting that *COPB2* was also involved in downstream TGF-β signaling. As TGF-β signaling was involved in tumor invasion, *COPB2* might induce the invasion of PCa cells through downstream TGF-β signaling pathways.

### NUPR1 is the Interaction Protein Downstream of COPB2 Expression

**Figure 8:**
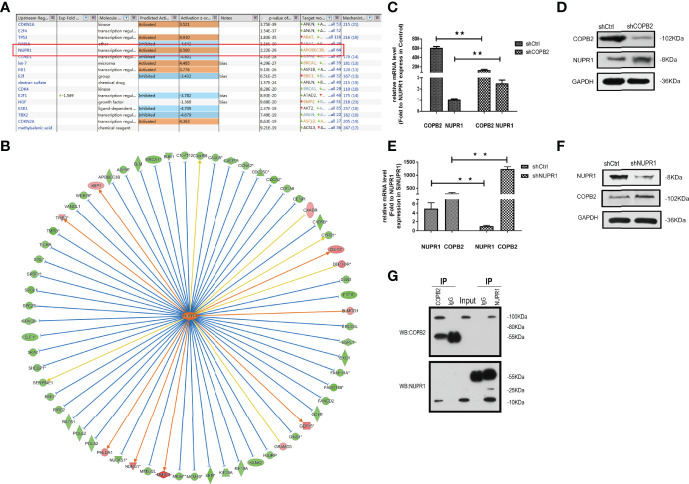
*NUPR1* is the interaction protein in the downstream of *COPB2*. **(A)** The most different downstream regulators after *COPB2* was downregulated, in which *NUPR1* was the most activated (*Z*-score = 6.500), *ESR1* was the most inhibited (*Z*-score = -4.709). **(B)** The interaction between *NUPR1* and its downstream regulators by IPA analysis. Orange line between the upstream regulator and gene expression of consistent activation state, the blue line shows the upstream consistent inhibition between regulators and gene expression of the state, and the yellow line shows the upstream regulator between gene expression of the state, gray line shows the state related data set does not exist and the expression of forecasting information. **(C, D)** The mRNA and protein expression of NUPR1 after *COPB2* knockdown *via* qRT-PCR and western blot. **(E, F)** The mRNA and protein expression of COPB2 after *NUPR1* knockdown *via* qRT-PCR and western blot. **(G)** *COPB2* can combine with *NUPR1* by Co-IP. **P < 0.01.

**Figure 9:**
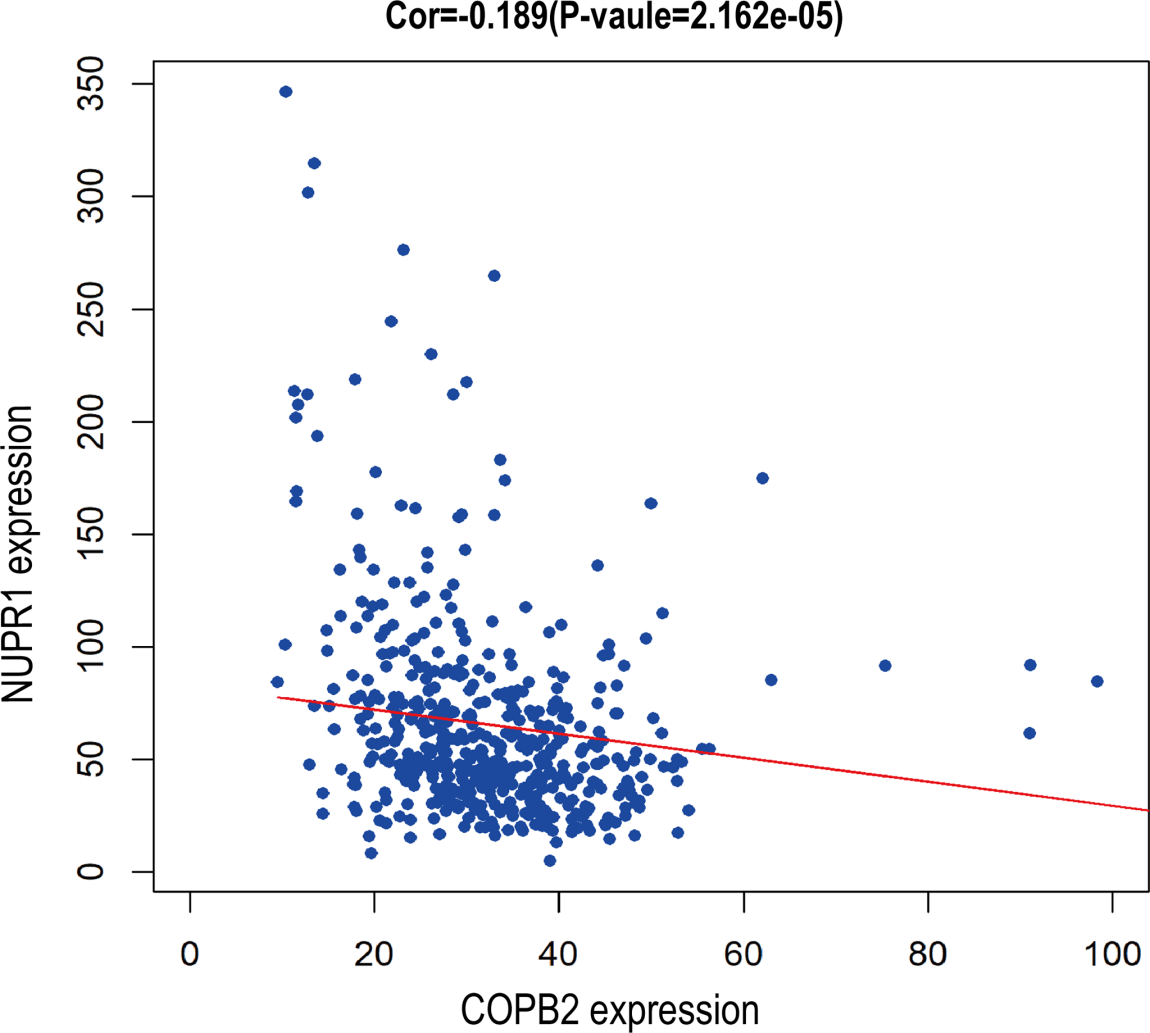
Bioassay showed negative correlation between *COPB2* and *NUPR1* expression.

When COPB2 expression was interfered in PC-3 cells, the whole gene expression profile was detected by GeneChip analysis, and the date were analyzed by bioinformatics and IPA. The results indicated that NUPR1 was the most activated protein (*Z*-score = 6.500), and its expression was most significantly upregulated after COPB2 expression was inhibited (**Figure 8A**). IPA bioassay revealed NUPR1 regulatory target genes (**Figure 8B**). In addition, the negative regulation of COPB2 and NUPR1 was verified in PC-3 cells. After *COPB2* knockdown, NUPR1 mRNA and protein expression was upregulated, as revealed *via* qRT-PCR and western blot, respectively (**Figures 8C, D**). In contrast, COPB2 expression was upregulated after *NUPR1* knockdown (**Figures 8E, F**). Then *COPB2* and *NUPR1* binding was verified in 293T cells. The Co-IP assay confirmed that the COPB2 antibody pulled down NUPR1 expression, whereas the NUPR1 antibody pulled down COPB2 expression, indicating that they were bound to each other (**Figure 8G**). Furthermore, the expression data of COPB2 and NUPR1 in PCa tissues were extracted from the TCGA database, and the relativity between COPB2 and NUPR1 expression was analyzed using the Pearson correlation coefficient. The results indicated that there was a negative correlation between COPB2 and NUPR1 expression in PCa tissues (*P* < 0.01) (**Figure 9**).

## Discussion

**Figure 10:**
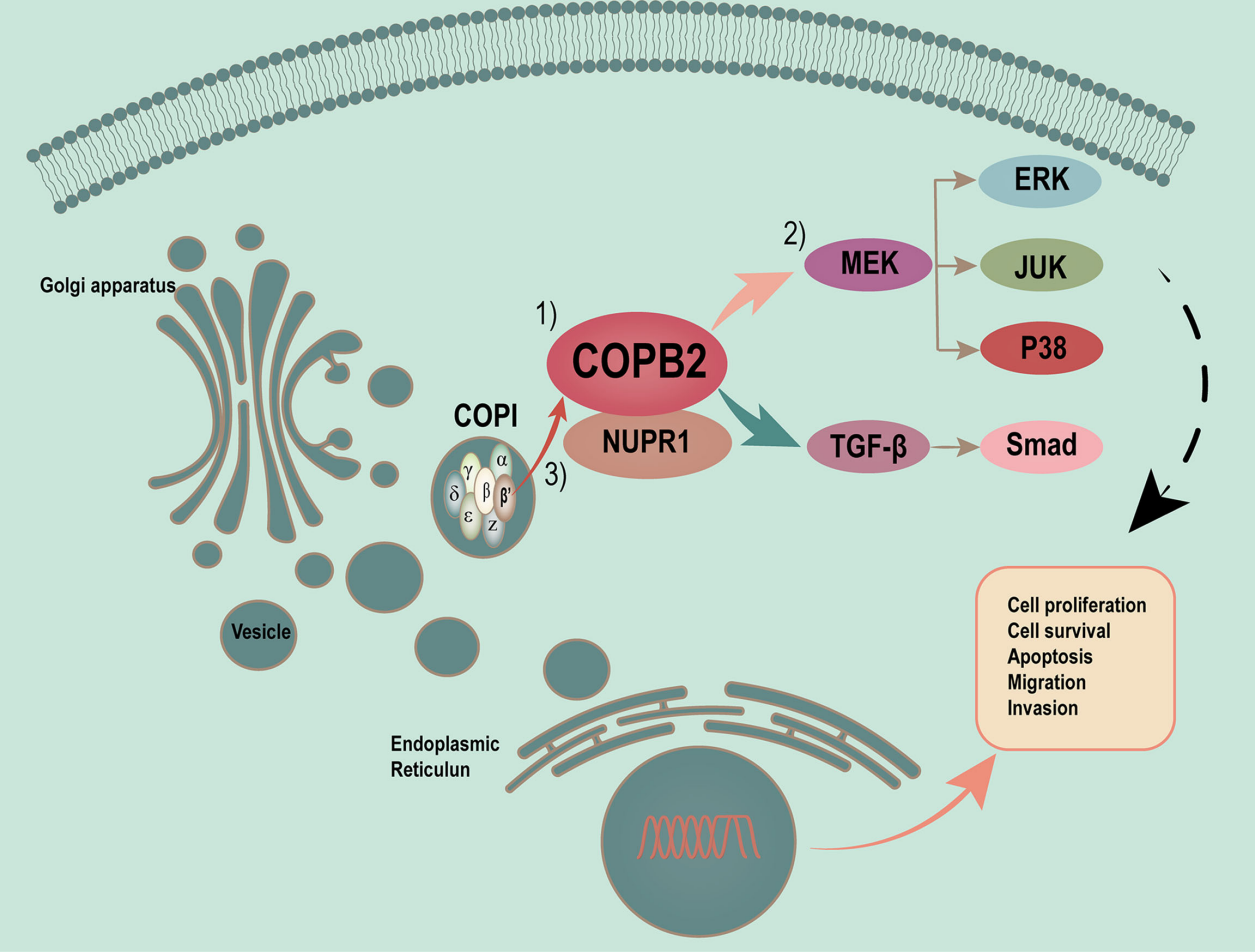
Proposed model of MAPK/TGF-β/COPB2/NUPR1 axis in control cell proliferation, migration and invasion in PCa cells. Vesicles plays a critical role in membrane transport between ER and Golgi apparatus. COPI-coated vesicles, which contain seven non-clathrin-coated vesicular coat subunits, play its function in the early secretory pathway. COPB2 is one of subunits involving in transport between ER and Golgi. After many researches have been done, we have identified the differential and functional COPB2 protein. On one hand, the *COPB2* can be as a molecular to be secreted to serum and be considered as a biomarker to detect some potential PCa patients. On the other hand, more important, *COPB2* can promote cell proliferation, migration and invasion *via* MAPK/TGF-β signaling pathway in PCa. The least but not the last, *COPB2* can interaction with *NUPR1*, which is a transcription factor associated with cancer development and advancement.

Vesicles play a critical role in membrane transport between the endoplasmic reticulum (ER) and Golgi apparatus (19, 20). COPI-coated vesicles, which contain seven non-clathrin-coated vesicular coat subunits, play a role in the early secretory pathway. COPB2 is one of subunits involving the transport between ER and Golgi (21–25). After so many researches, the differential and functional COPB2 protein was identified. *COPB2* is involved in material transport, energy metabolism as a vesicular envelope protein and tumor progression (15, 26–28). In the present study, a model of MAPK/TGF-β/COPB2/NUPR1 axis in control of cell proliferation, migration, and invasion in PCa cells was proposed (**Figure 10**).

COPB2 can be secreted as a molecule in serum and considered as a biomarker to detect potential PCa patients. First, *COPB2* was associated with clinical characteristics and biochemical recurrence, and patients with high COPB2 expression were likely to have high Gleason scores and biochemical recurrence rates after RP. The results of proliferation- and invasion-related experiments showed that interfering with COPB2 notably restrained the proliferation and invasion ability of PCa cells, indicating that COPB2 was an oncoprotein. Sudo et al. (29) first confirmed that siRNA interference with *COPB2* could inhibit cell proliferation and promote apoptosis in a mouse model of malignant mesothelioma. Furthermore, it was reported that *COPB2* affected the cell cycle by regulating p21 and cyclin A, promoted the proliferation of colon cancer cells ultimately (30). Li et al. (31) confirmed that knock-downing COPB2 markedly inhibited the proliferation of bile duct cancer cells and promoted apoptosis in the G1 phase by blocking the cell cycle. The aforementioned studies corroborated this study’s results, which jointly stated that *COPB2* was an oncogene, which was of great significance for further research.

Moreover, COPB2 promoted cell proliferation, and invasion *via* MAPK/TGF-β signaling pathway in PCa. The expression spectrum GeneChip analysis combined with IPA were used to analyze the downstream signaling pathway in the initial screening and revealed the involvement of downstream MAPK/TGF-β signaling in the proliferation and invasion of PCa.

COPB2 interacted with NUPR1, which was a transcription factor associated with cancer development and advancement; *NUPR1* produced a marked effect in cellular stress response and participated in tumor metastasis (32). *NUPR1* was first found in a rat acute pancreatitis model and was afterwards found to be high-expressed in human metastatic breast cancer cells and identified as com-1 (33). Knocking down *NUPR1* in liver cancer, pancreatic cancer, glioma, and lung cancer inhibited cell proliferation, migration, and invasion and EMT transformation; it also promoted apoptosis, increased tumor drug sensitivity, and participated in MAPK and TGF-β pathways—MAPK and TGF-β were potential oncoproteins (34–36). However, in some types of tumors, *NUPR1* acted as a tumor suppressor gene. In a previous study, the expression level of *NUPR1* was negatively correlated with the grade of colon cancer, and the expression of *NUPR1* was lower in high-grade colon cancer tissues than in normal tissues (37). *NUPR1* was significantly under-expressed in PCa tissues, with knocking down *NUPR1* accelerating cell growth and enhancing infiltration capacity. Overexpression of *NUPR1* reversed phenotype and significantly inhibited the growth of Nude-mice subcutaneous graft tumors (38). In this study, *COPB2* and *NUPR1* exhibited a negative regulatory relationship, which expanded the understanding of the *COPB2* mechanism. Meanwhile, there weren’t retrieving relevant reports on the mutual regulation of *COPB2* and *NUPR1*. Therefore, further in-depth studies of the role and mechanism in the PCa progression is of utmost importance.

There are also a few limitations in this study. Owing to the limited number of clinical samples, false-positive or false-negative conclusions may have occurred, the results need to be supplemented by subsequent experiments. Subcutaneous tumorigenesis in Nude mice was used in the *in vivo* experiment, which lacked the specificity of PCa; the *in situ* model of PCa should be established in a follow-up experiment, that will be more convincing. In future studies, the select appropriate pathway inhibitors and protein markers should be chosen for functional tests, the positive and negative feedback experiments should be selected to verify whether COPB2 further participates in the biological functions of PCa through MAPK/TGF-β signaling pathway. Finally, the relationships or functions (such as co-location, co-expression, rescue experiments) between *NUPR1* and *COPB2* should be carried out, and also the subsequent mechanisms need to be further studied.

## Conclusion

The differential functional protein COPB2 is expected to become a new biomarker for PCa diagnosis and monitoring, and studying it may provide a theoretical basis for identifying effective drug intervention targets through in-depth mechanistic studies.

## Data Availability Statement

Publicly available datasets were analyzed in this study. This data can be found here.

http://tcga-data.nci.nih.gov/tcga/

http://www.ncbi.nlm.nih.gov/geo/

http://www.r-project.org

## Ethics Statement

The project was authorized by the Ethics Review Committees of the Affiliated Hospital of Jiangnan University, Affiliated Changzhou No. 2 Hospital of Nanjing Medical University, and Huashan Hospital of Fudan University. (Batch number of ethical approval document: LS202128). Written informed consent to participate in this study was provided by the participants’ legal guardian/next of kin. The animal study was reviewed and approved by the Ethics Review Committees of Jiangnan University (approval number: JN.No20201030c0561225[274]).

## Author Contributions

YF, CS, and LFZ were the co-contributor in writing the manuscript. HW and HZ performed the literature search. YC made guidance on the grammar of the article. LJZ, GX, and YM made substantial contributions to the design of the manuscript and revised it critically for important intellectual content. All authors contributed to the article and approved the submitted version.

## Funding

This work was supported by the National Natural Science Foundation (Number: 81802576, 81902565, 81372316), Wuxi Commission of Health and Family Planning (Number: T202024, J202012, Z202011), The Science and Technology Development Fund of Wuxi (Number: N20202021), Jiangnan University Wuxi School of Medicine (No. 1286010242190070), Wuxi Taihu Lake Talent Plan, Supports for Leading Talents in Medical and Health Profession, and Top Talent Support Program for Young and Middle-aged People of Wuxi Health Committee (Number: BJ2020061).

## Conflict of Interest

The authors declare that the research was conducted in the absence of any commercial or financial relationships that could be construed as a potential conflict of interest.

## Publisher’s Note

All claims expressed in this article are solely those of the authors and do not necessarily represent those of their affiliated organizations, or those of the publisher, the editors and the reviewers. Any product that may be evaluated in this article, or claim that may be made by its manufacturer, is not guaranteed or endorsed by the publisher.
