# Supplementary material for: Upregulation of COPB2 Promotes Prostate Cancer Proliferation and Invasion Through the MAPK/TGF-β Signaling Pathway
Source: Front Oncol. 2022 May 6;12:865317. doi: 10.3389/fonc.2022.865317 (PMC9120942; doi:10.3389/fonc.2022.865317)

***Supplementary Material***

**Upregulation of COPB2 promotes** **prostate cancer proliferation and invasion through** **the** **MAPK/TGF-β signaling pathway**

**FIGURE LEGENDS**

**Supplementary Figure 1** | Disease and function heat map showed the relationships between differentially expressed genes and functions in related diseases. Orange means *Z*-score > 0, blue means *Z*-score < 0, gray means that there was no *Z*-score value; *Z*-score > 2 means that the function was significantly activated, and *Z*-score < -2 means that the function was significantly inhibited.

**Supplementary Figure 2** | Disease and function histogram showed the enrichment of differentially expressed genes in disease and functional categories. All diseases and functions are sorted using [-Log(*P*-value) Transformation].

**Supplementary Figure 3** | The upstream regulatory sub-network diagram showed the enrichment of differentially expressed genes in the classical signaling pathways [-Log(*P*-value) Transformation]. Orange signaling pathways indicate *Z*-Score >0, while blue signaling pathways indicate *Z*-Score < 0. *Z*-score > 2 indicates that the pathways are significantly activated, and *Z*-Score < -2 indicate that the pathways are significantly inhibited. Ratio represents the ratio of the number of differential genes in this signaling pathways to the number of all genes in the signaling pathways.

**Supplementary Figure 4** | The regulatory effect network diagram showed the interaction between genes/regulators and corresponding functions in the dataset. IPA performed interaction analysis and functional annotation for differential genes. The regulators (ATF4, CDKN1A, CDKN2A, E2f, E2F1, EP400, FOXO1, let-7, MITF, MYC, NLRP3, NUPR1, RABL6, RB1, TBX2, TP53) activate congenital malformation of brain and neural tube defect by the genes (ASPM,BLM, BRCA1, BRCA2, CASC5, CCNA2, CCNE2, CDC25C, CDC6, CDCA2, CDK1, CDT1, CEBPD, CHEK1, CIT, CTNNBIP1, CXADR, CYCS, CYR61, DDIT3, DHFR, DLGAP5, DTL, E2F8, ERCC1, ESPL1, EXOSC8, FANCD2, FKBP1A, GDF15, HMOX1, KANK2, KIAA0101, KIF18A, MAD2L1, MCM10, MCM4, MELK, MMS22L, MPDZ, MTHFR, NCAPG, NCAPG2, NDRG1, NEK2, NUSAP1, ORC1, ORC6, PLSCR1, RAD51, RFC3, SGOL1, SMAD2, STIL, TACC3, TEAD1, TGFBR2, TOPBP1, TP63, TPD52L1, TYMS, VANGL1, VRK1, WDHD1, XBP1) and inhibit alignment of chromosomes, association of chromosome components, G2/M phase, homologous recombination of cells, interphase of tumor cell lines and size of embryo.

Supplementary Figure 1


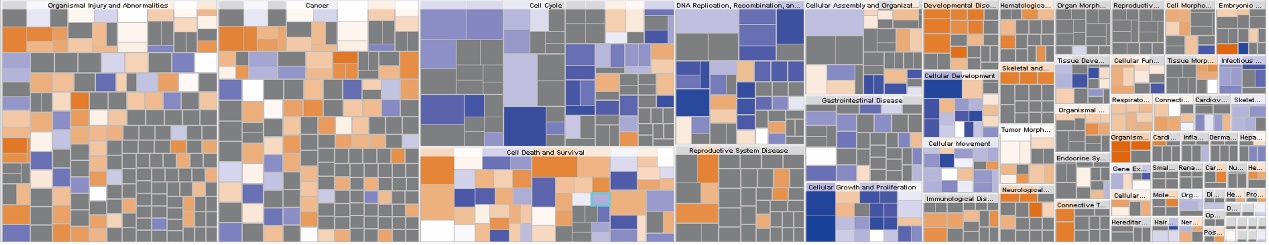


Supplementary Figure 2
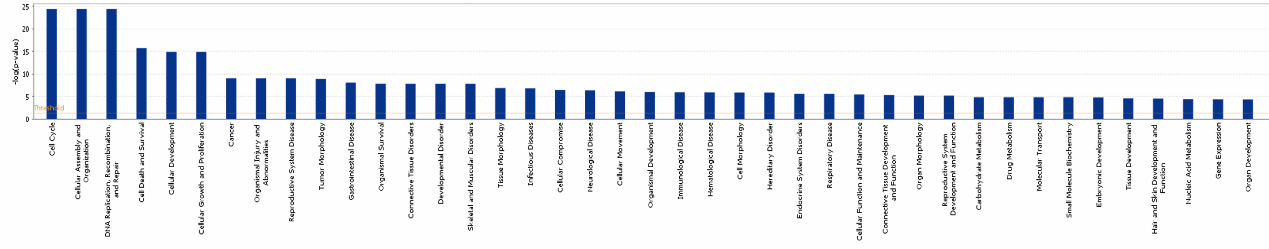


Supplementary Figure 3
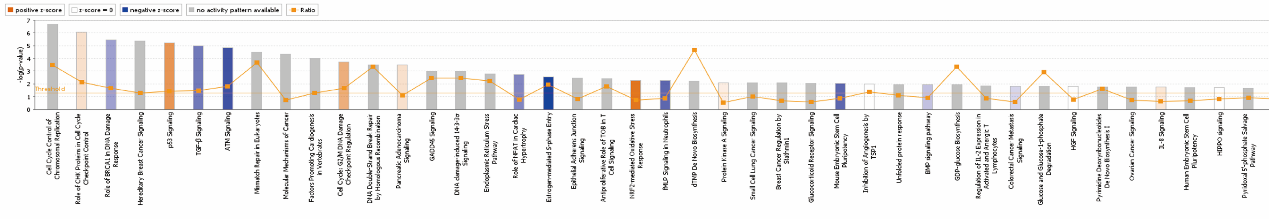


Supplementary Figure 4


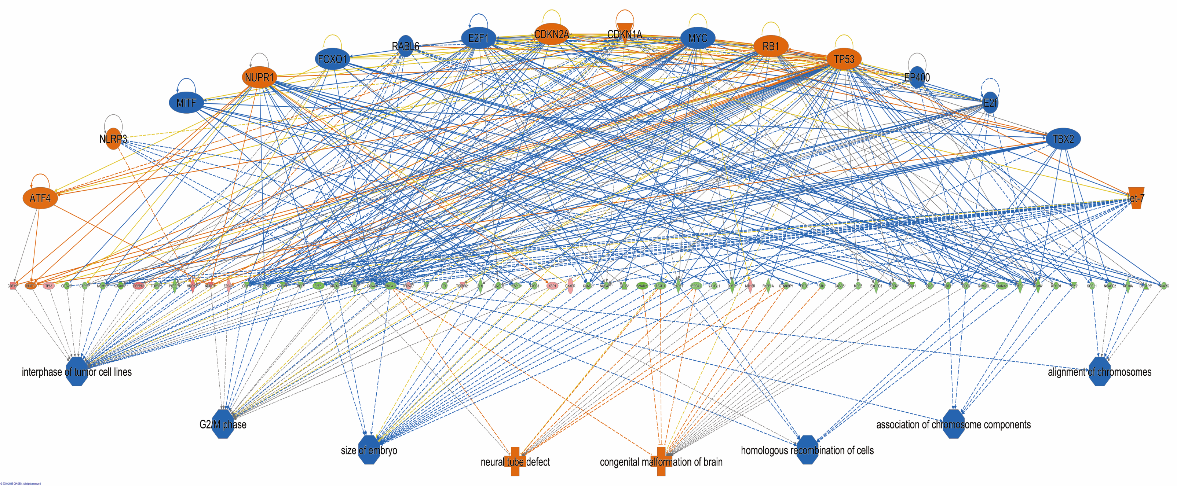

Supplement: Supplementary file 1 [file DataSheet_1.docx]
