# Supplementary material for: Upregulation of COPB2 Promotes Prostate Cancer Proliferation and Invasion Through the MAPK/TGF-β Signaling Pathway
Source: Front Oncol. 2022 May 6;12:865317. doi: 10.3389/fonc.2022.865317 (PMC9120942; doi:10.3389/fonc.2022.865317)
Supplement: Supplementary file 2 [file DataSheet_2.pdf]

Western Blot Report

1. Gene Information

E-cadherin Vimentin FN1 N-cadherin Snail

2. Experiment

1） Antibody Information

Primary antibody：

Antibody	Origin of Species	Company	Number	Dilution Rate	Size	Positive control sample
E-cadherin	Mouse	CST	#14472	1:1000	135 kDa	MCF-7
Vimentin	Rabbit	CST	#5741	1:1000	57 kDa	HeLa NIH/3T3 C6
FN1	Mouse	abcam	ab6328	1:400	25kDa-250 kDa	Unknown
N-cadherin	Rabbit	CST	#13116	1:1000	140 kDa	MCF-7
Snail	Mouse	CST	#3895	1:1000	29 kDa	NIH/3T3
GAPDH	Mouse	Santa Cruz	SC-32233	1：2000	36 kDa	No

Second antibody：

Antibody	Company	Number	Dilution Rate
rabbit IgG	Santa Cruz	sc-2004	1:5000
mouse IgG	Santa Cruz	sc-2005	1:5000

2） Experiment Paramete of Western blot

SDS-PAGE Separation gel concentration：8/ 10 %

Protein loading quantity：20 μg

Chromogenic system： ECL

3. Experimental Procedure（Please refer to the literature[1-2]）

4. Result

1） Result of western blot

Antibody	images

**E-cadherin**<br>Dilution Rate：1:500<br>135 kDa<br>**Vimentin**<br>Dilution Rate：1:500<br>57 kDa<br>(DU 145 cell)	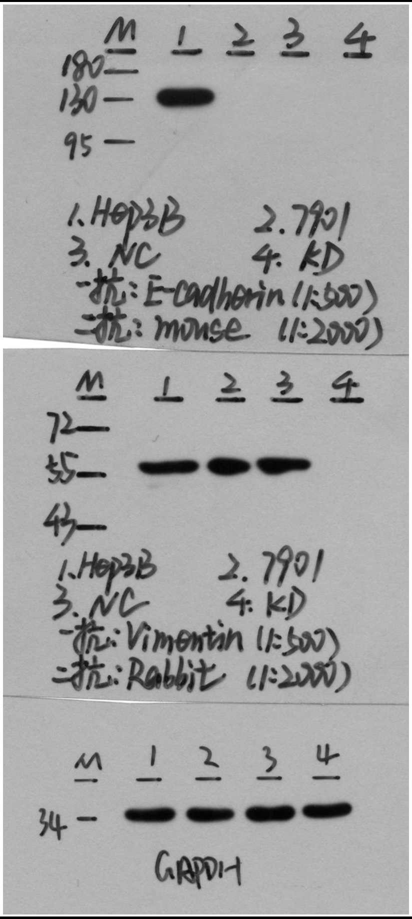
**E-cadherin**<br>Dilution Rate：1:500<br>135 kDa<br>**Vimentin**<br>Dilution Rate：1:500<br>57 kDa<br>(PC-3 cell)	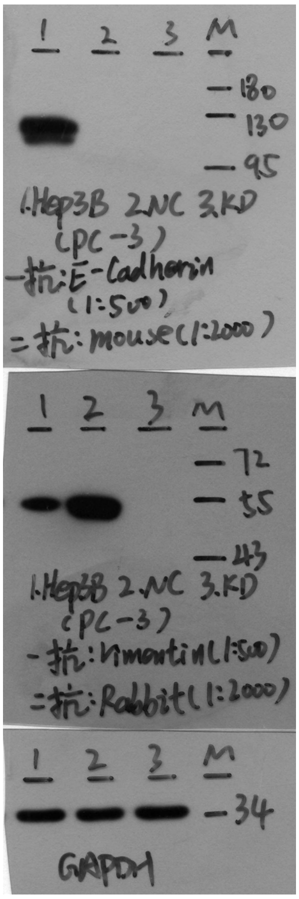

<table>
<tr>
<td>FN1

Dilution Rate：1:500

25kDa-250 kDa
(DU 145 cell)</td>
<td>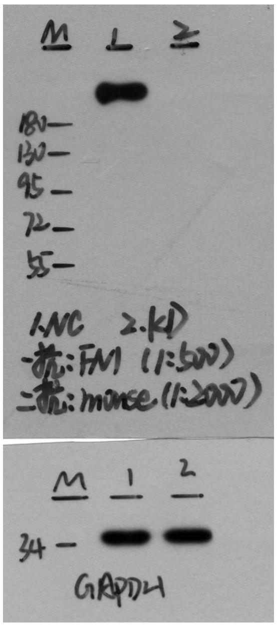
</td>
</tr>
<tr>
<td>FN1

Dilution Rate：1:500

25kDa-250 kDa
(PC-3 cell)</td>
<td>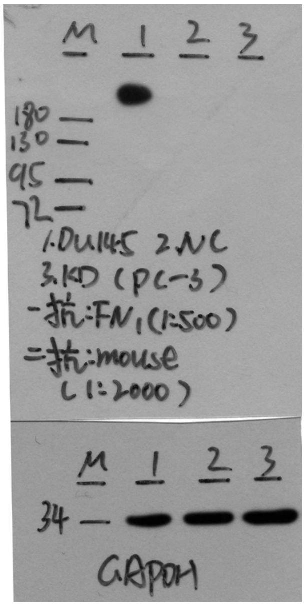
</td>
</tr>
</table>

N-cadherin<br>Dilution Rate：1:200<br>100 kDa<br>Snail<br>Dilution Rate：1:1000<br>29 kDa<br>(DU 145 cell)	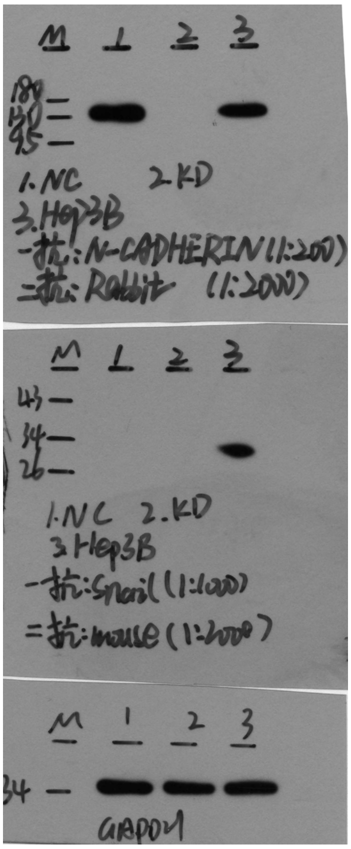
N-cadherin<br>Dilution Rate：1:200<br>100 kDa<br>Snail<br>Dilution Rate：1:1000<br>29 kDa<br>(PC-3 cell)	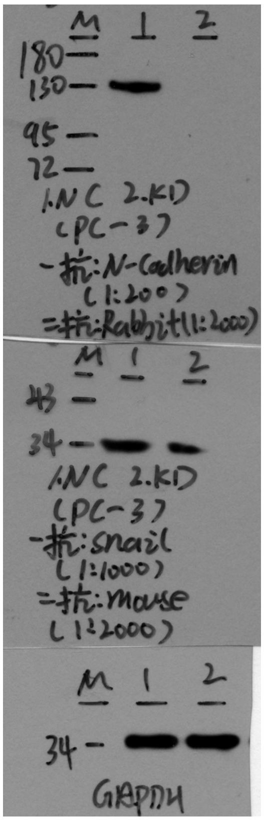

Note:

Sample	Description

M：Marker	The strip size is shown
cell samples	PC-3，DU 145
Positive control sample	Hep3B，SGC-7901，DU 145

2）Conclusion：

Antibody	Description
E-cadherin	No target band was detected in DU 145 cell, but the Hep3B cell was detected；<br>No target band was detected in PC-3 cell, but the Hep3B cell was detected.
Vimentin	The target band was detected in DU 145 cell, and shCOPB2 group was significantly down-regulated relative to shCtrl group.<br>The target band was detected in PC-3 cell, and shCOPB2 group was significantly down-regulated relative to shCtrl group.
FN1	The target band was detected in DU 145 cell, and shCOPB2 group was significantly down-regulated relative to shCtrl group.<br>No target band was detected in PC-3 cell, but the DU 145 cell was detected.
N-cadherin	The target band was detected in DU 145 cell, and shCOPB2 group was significantly down-regulated relative to shCtrl group.<br>The target band was detected in PC-3 cell, and shCOPB2 group was significantly down-regulated relative to shCtrl group.
Snail	No target band was detected in DU 145 cell, but the Hep3B cell was detected.<br>The target band was detected in PC-3 cell, and shCOPB2 group was significantly down-regulated relative to shCtrl group.

[1]Mi Y, Sun C, Wei B, Sun F, Guo Y, Hu Q, et al. Coatomer Subunit Beta 2 (Copb2), Identified by Label-Free Quantitative Proteomics, Regulates Cell Proliferation and Apoptosis in Human Prostate Carcinoma Cells. *Biochem Biophys Res Commun* (2018) 495(1):473-80. doi: 10.1016/j.bbrc.2017.11.040.

[2]Mi Y, Yu M, Zhang L, Sun C, Wei B, Ding W, et al. Copb2 Is Upregulated in Prostate Cancer and Regulates Pc-3 Cell Proliferation, Cell Cycle, and Apoptosis. *Arch Med Res* (2016) 47(6):411-8. doi: 10.1016/j.arcmed.2016.09.005.
