# Supplementary figures and images for: Upregulation of COPB2 Promotes Prostate Cancer Proliferation and Invasion Through the MAPK/TGF-β Signaling Pathway
Source: Front Oncol. 2022 May 6;12:865317. doi: 10.3389/fonc.2022.865317 (PMC9120942; doi:10.3389/fonc.2022.865317)

Figure 2A

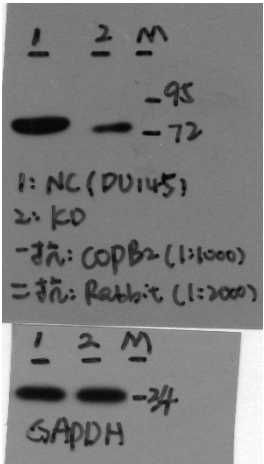

Figure 3C

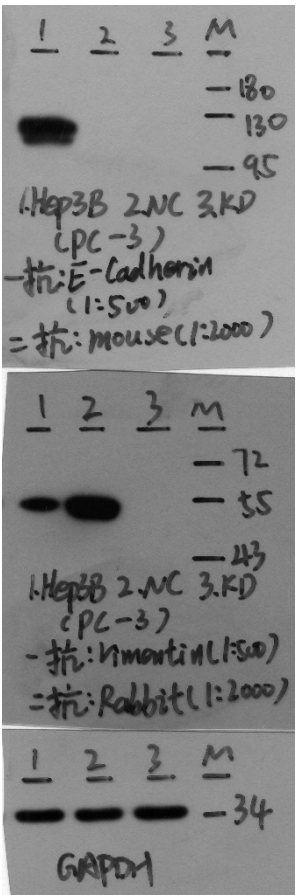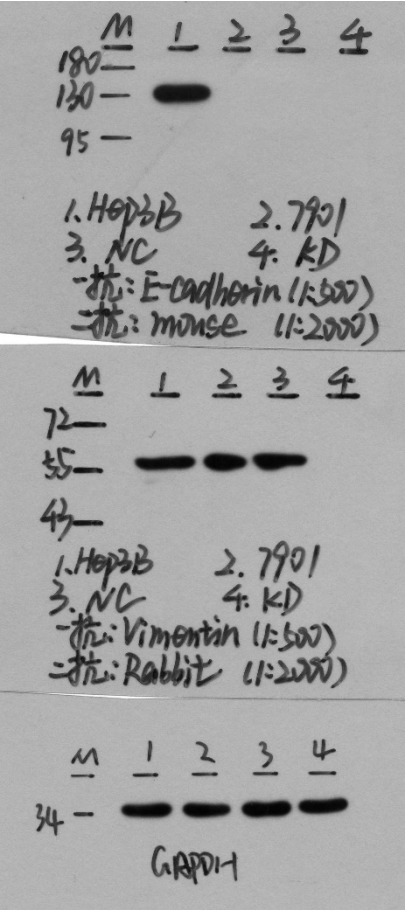

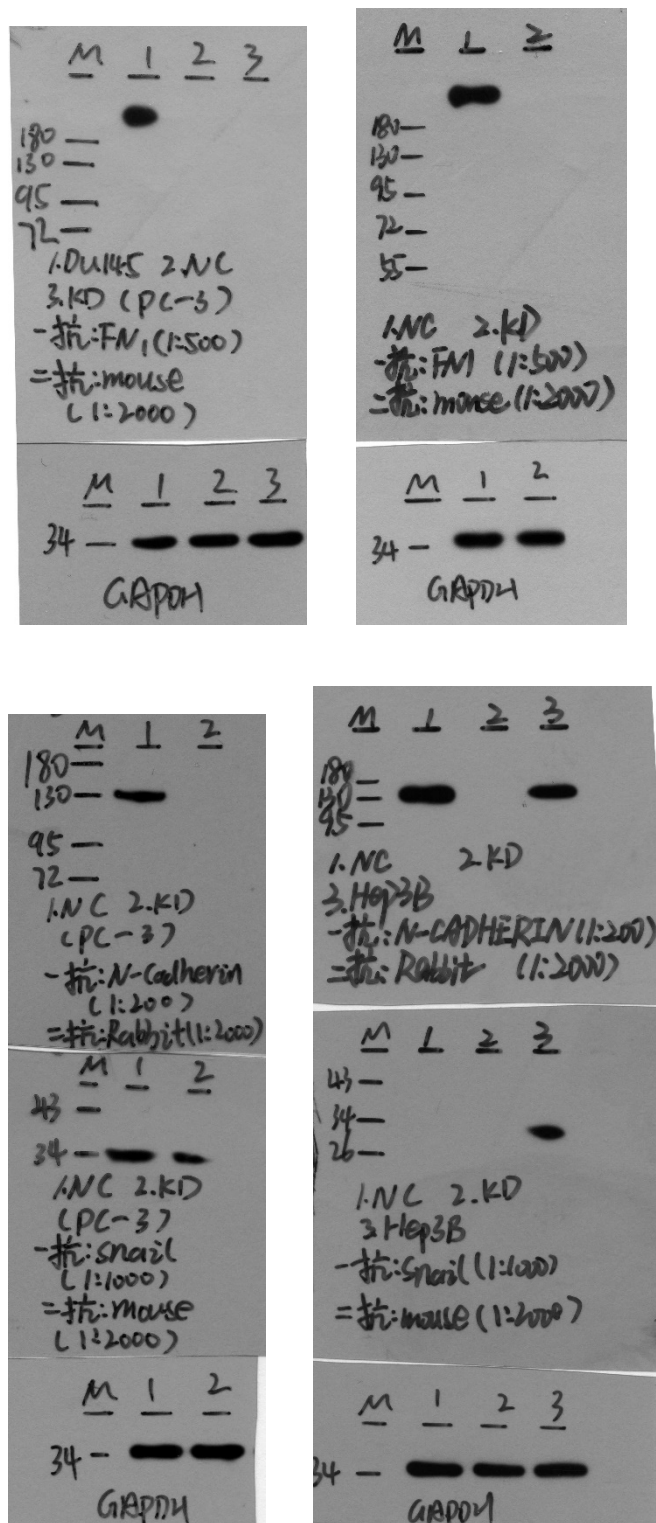

Figure 5

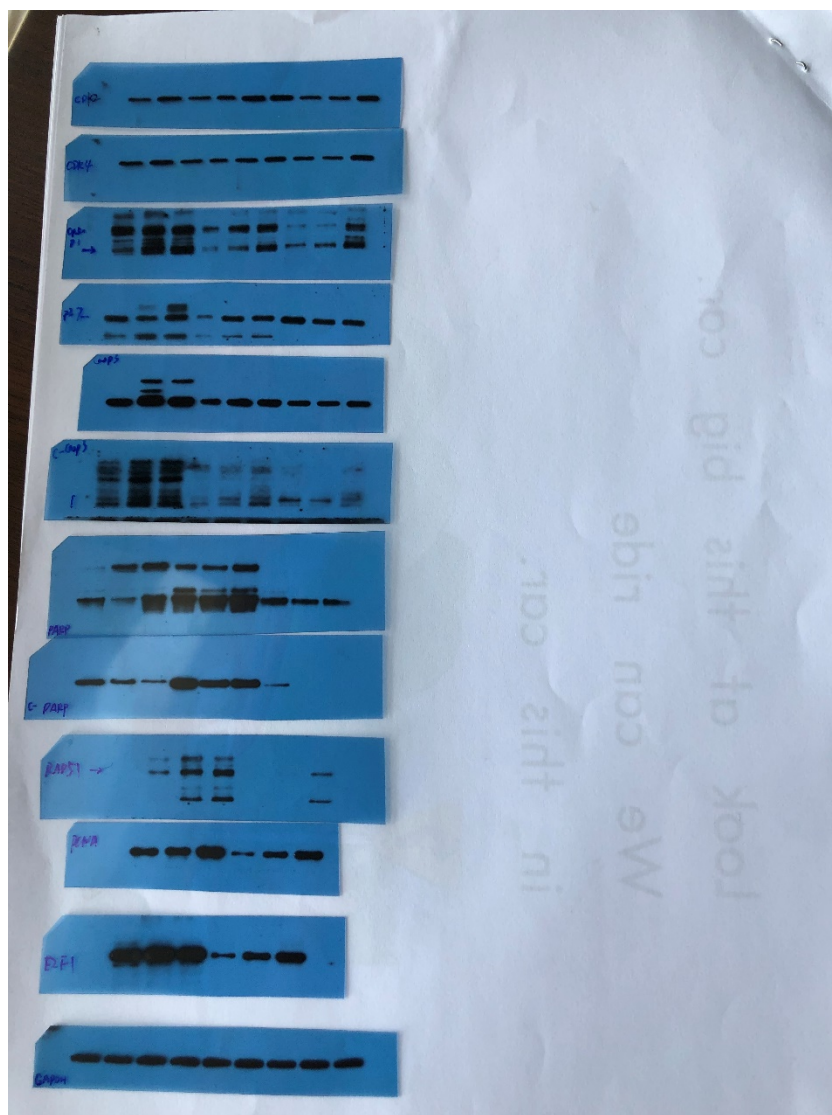

Figure 7

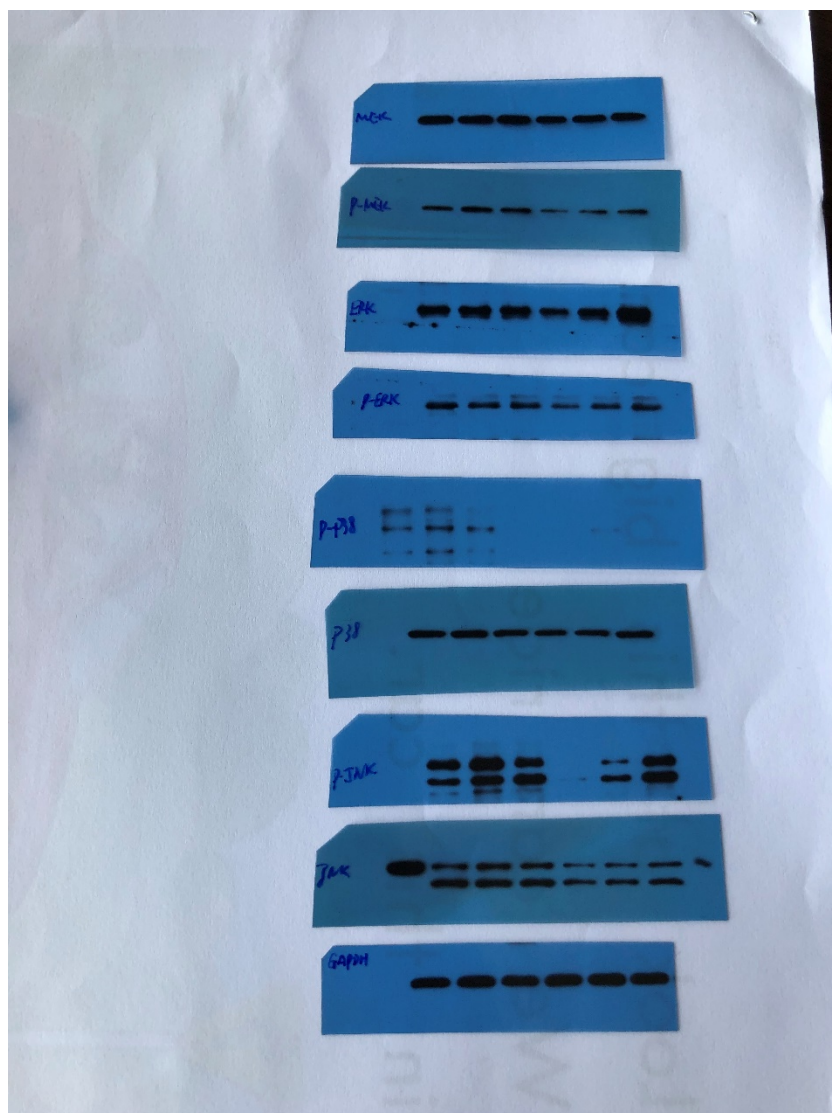

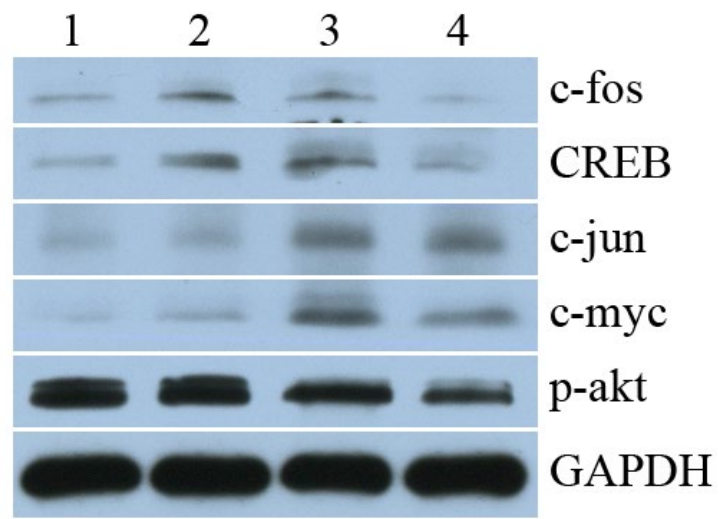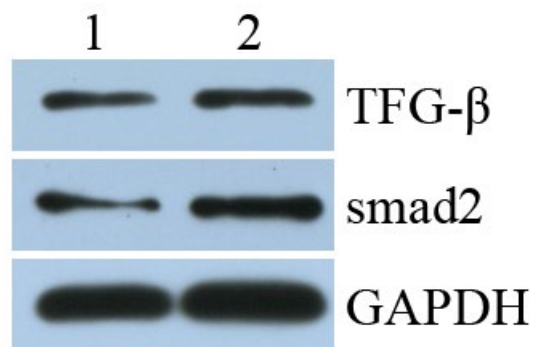

Figure 8

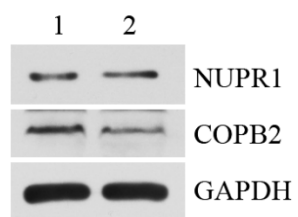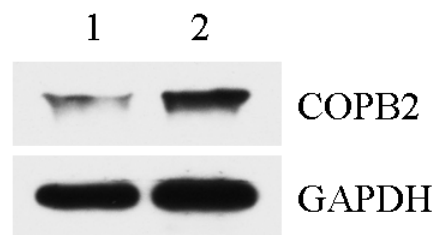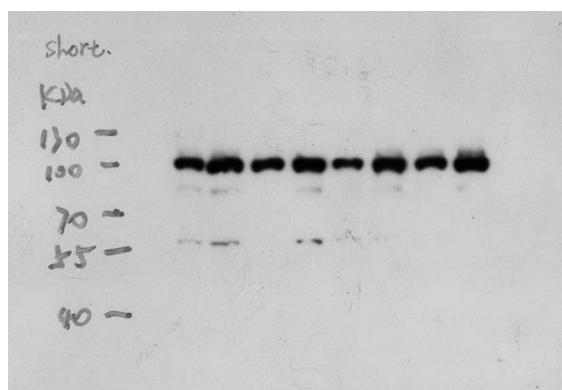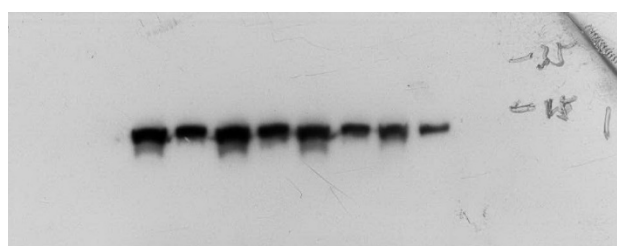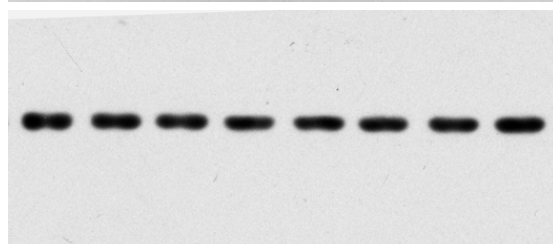

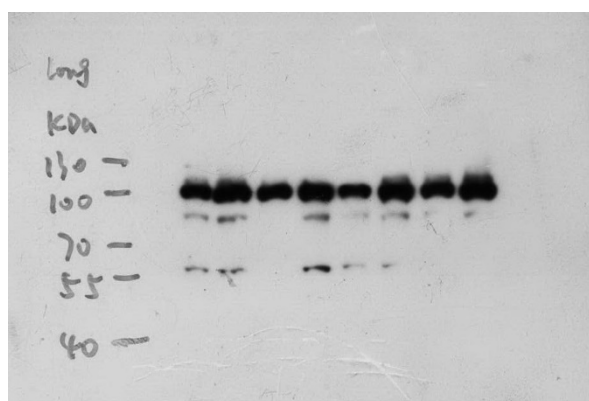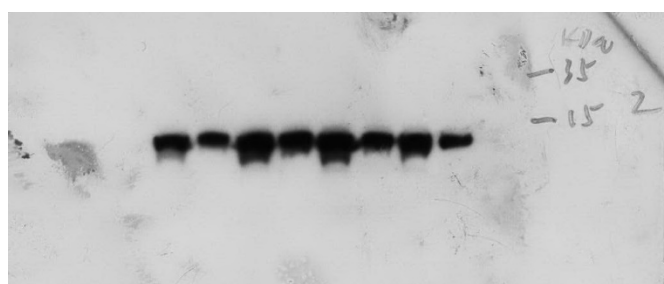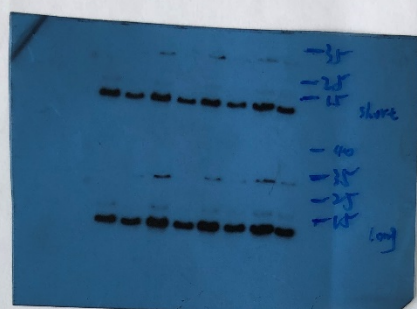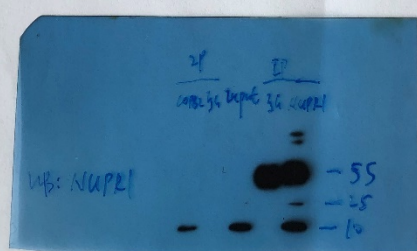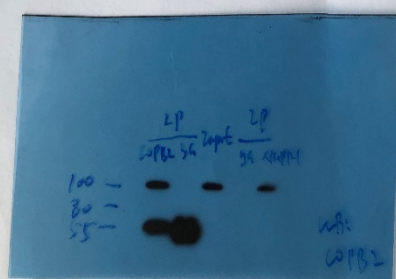

Supplement: Supplementary file 4 [file DataSheet_4.pdf]
